# A bibliometric study on clinical research in neonatal encephalopathy

**DOI:** 10.3389/fped.2024.1403671

**Published:** 2024-11-01

**Authors:** Shujun Tan, Gulizuohere Alimujiang, Nuerya Rejiafu

**Affiliations:** ^1^ Neonatal Center, Children's Hospital of Xinjiang Uygur Autonomous Region, Urumqi, China; ^2^ Neonatal Center, Xinjiang Hospital of Beijing Children's Hospital, Children's Hospital of Xinjiang Uygur Autonomous Region, Urumqi, China; ^3^ Neonatal Center, The Seventh People's Hospital of Xinjiang Uygur Autonomous Region, Urumqi, China; ^4^ Graduate School, Xinjiang Medical University, Urumqi, China

**Keywords:** neonatal encephalopathy, bibliometric, visualization, research trends, neonatal

## Abstract

This research presents a comprehensive review of studies on neonatal encephalopathy conducted between 2005 and 2024, utilizing knowledge graph analysis through CiteSpace and VOSviewer software. A search of the Web of Science core database identified 893 articles, with the United States emerging as a prominent contributor in terms of publication volume. Key co-occurrence keywords identified include “Hypoxic-ischemic encephalopathy”, “Neonatal encephalopathy”, and “Therapeutic hypothermia”. Notable contributors, such as Seetha Shankaran and Floris Groenendaal, have significantly advanced research in this area. Leading institutions in this field include the University of Washington, while the journal Pediatrics is recognized as a leading publication in the domain of neonatal encephalopathy. These findings provide a solid foundation for guiding future research endeavors.

## 1 Introduction

The neonatal mortality rate serves as a vital metric for evaluating the health and medical standards of a country or region (1). Estimates indicate that roughly 2.5 million neonatal deaths transpire worldwide annually (2). Neonatal encephalopathy (NE), which impacts infants born after 35 weeks of gestation, disrupts neurological function (3). The main sign is an altered consciousness level or quality, alongside potential indicators such as seizures, problems with the heart and lungs, or abnormal reflex actions (3–5). NE affects between 2 and 8 out of every 1,000 livebirths, posing a considerable risk of death or severe impairment (6). It significantly contributes to global mortality and morbidity, affecting about 1.4 million infants each year and standing as the third most common cause of death among children under 5 years old (7). NE also substantially influences long-term neurological morbidity globally, contributing to a 35% surge in childhood disabilities over the past two decades, owing to better survival rates (8). NE survivors may endure lasting impacts such as cerebral palsy (CP), overall developmental delays (GDD), impairments in vision and hearing, and seizure disorders (9).

Therapeutic hypothermia, commonly referred to as HT, is widely acknowledged as the most successful method for protecting the infant brain in cases of encephalopathy (10, 11). This procedure involves decreasing the body temperature of high-risk newborns to 33.5°C for a period of 72 h. Despite a 25% decrease in the relative risk of mortality, almost half (46%) of the infants displayed unfavorable results in scientific inquiries (11–13). In recent times, there has been an increasing focus on the use of hypothermia treatment (HT) in intensive care environments to enhance newborn brain health. Nonetheless, there has been minimal progress in improving outcomes (12).

NE significantly impacts the physical and cognitive development of infants (14). It is crucial for the medical community to prioritize research efforts to enhance treatment methods for this condition. Increased research endeavors will result in better medical resources and a broader range of treatment options, ultimately enhancing the overall quality of life for the population. Moreover, advancements in healthcare will decrease the occurrence of complications and alleviate the financial burden on families due to illness. This bibliometric study concentrates on NE to provide further insights into this significant research field.

The origins of bibliometrics can be traced back to the early 20th century. In 1917, Cole and Eales conducted a groundbreaking study that introduced the concept of bibliometrics by analyzing scientific activity patterns in the field of anatomy (15). Building upon this research, Lotka's work in 1926 further explored scientific productivity, ultimately leading to the formulation of Lotka's productivity law (16). This law emphasized that a small group of authors were responsible for the majority of published articles, while the majority of authors only contributed a few articles. These initial studies laid a solid foundation for the development of bibliometrics.

In recent times, there has been a significant rise in the amount of literature concerning NE. Nonetheless, there is still a notable absence of thorough analysis when it comes to publication trends in this area. Bibliometrics, a multidisciplinary field that includes mathematics, statistics, linguistics, and other related fields, is commonly used to assess pertinent literature within specific medical domains (17–20). Several bibliometric studies have proven its efficacy (21–23). Through careful analysis and the organization of information in a systematic way, bibliometrics allows for the evaluation of research quality, the identification of crucial research subjects, and the recognition of emerging patterns (24). Moreover, it has the capability to establish relationships between studies, predict future research trends, offer new perspectives to researchers, and enhance the effectiveness of scientific inquiries (25–27). High-quality bibliometric studies form a strong basis for advancing the field in innovative and influential methods. These types of research provide scholars with a comprehensive grasp of the topic, help pinpoint areas where information is lacking, and stimulate fresh research concepts. Additionally, bibliometric assessment acts as a valuable aid for conducting literature reviews (28).

The online database Web of Science (WOS) provides valuable data for conducting bibliometric analysis and can be utilized in conjunction with tools like VOSviewer and Citespace for in-depth examination. This research focuses on conducting a comprehensive analysis of numerous studies concerning neonatal asphyxia from a broad perspective using bibliometric techniques. By understanding the fundamentals of bibliometric analysis, this study aims to suggest potential research pathways that align with associated research fields. The research examines the following inquiries: Q1. Which authors exhibit the highest productivity in this area? Q2. What keywords and co-occurrence networks are trending, highlighting the current research topics?

## 2 Materials and methods

### 2.1 Data source and search strategy

**Figure 1:**
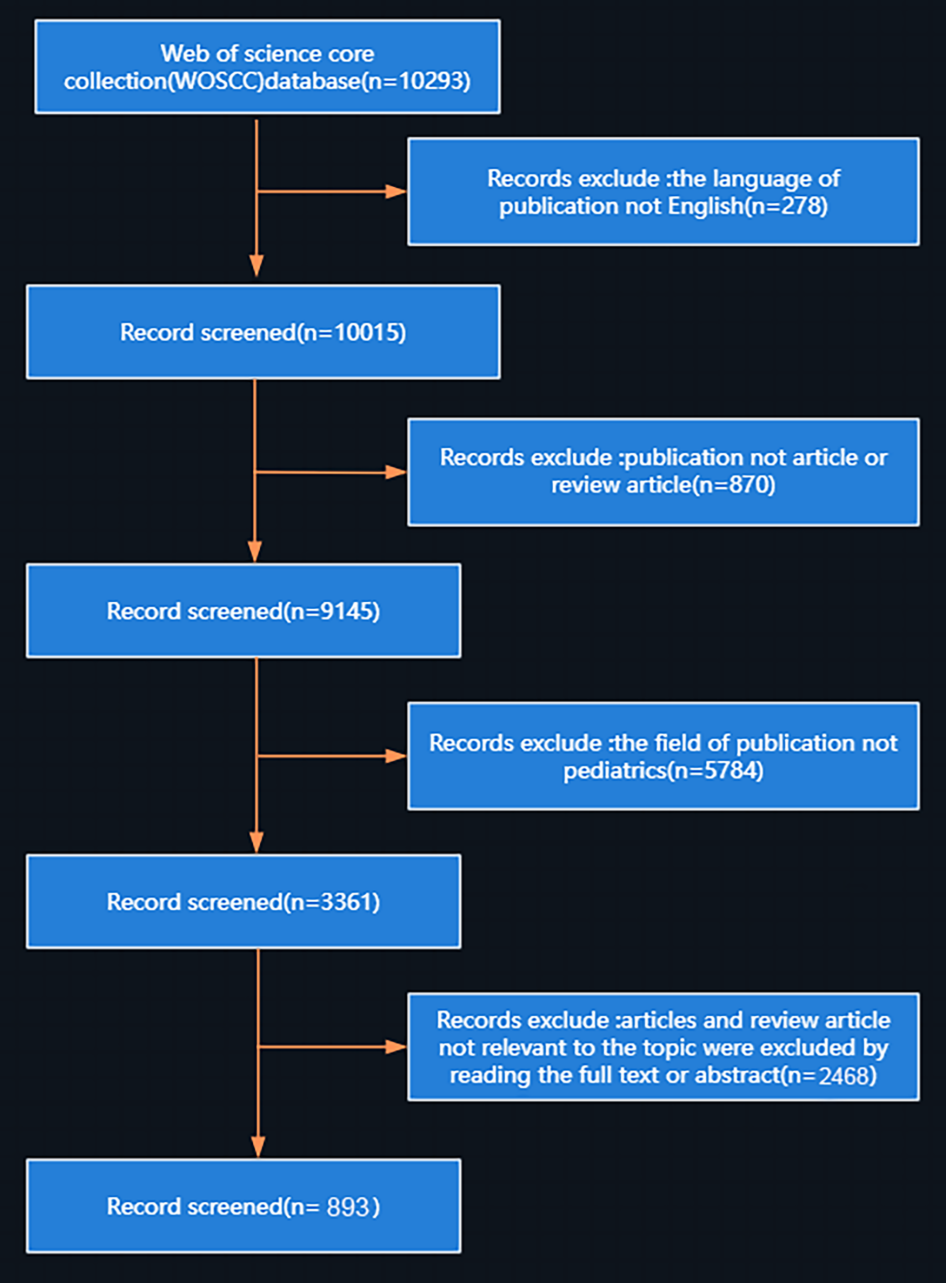
Flowchart of study enrollment.

This study utilizes the Web of Science Core Collection as its primary data source, a reputable digital database highly regarded for bibliometric analysis (29, 30). Data collection for the study was conducted on June 6, 2024, referencing Figure 1 and Table 1 to search for both articles and review articles on NE. This search also included meta-analyses and systematic reviews. Employing search terms such as “neonatal encephalopathy”, “asphyxia neonatorum”, “birth asphyxia”, “perinatal asphyxia”, and “hypoxic-ischaemic encephalopathy” within the advanced search interface, we targeted English articles and reviews published between January 1, 2005, and May 31, 2024. The search specifically focused on articles and reviews within the field of pediatrics. Initially, 3,361 potentially relevant articles were identified. However, the compilation of literature may include content that appears pertinent but is actually unrelated. Therefore, to ensure that the literature examined closely aligns with the research topic, a manual screening process is often necessary following the initial search. During this initial phase, materials deemed irrelevant are excluded based on their titles and abstracts, necessitating the involvement of two members of the research team. Subsequently, a detailed review of the complete texts is conducted to accurately identify the essential literature for the study. To execute the second stage of manual screening systematically and impartially, any disagreements among the researchers are resolved through discussion. After a thorough screening process and the elimination of irrelevant literature, a total of 893 documents were identified as suitable for further exploration in relevant fields.

**Table 1:** Summary of data source and selection.

Summary of data source and selection	
Category	Specific standard requirements
Research database	Web of science core collection
Searching period	January 1, 2005 to May 31, 2024
Language	“English”
Searching keywords	TS = (“neonatal encephalopathy” OR “asphyxia neonatorum” OR “birth asphyxia” OR “perinatal asphyxia” OR “hypoxic-ischaemic encephalopathy”)
Document types	“Article” OR “Review Article”
Data extraction	Export with full records and cited references in plain text format
Sample size	893

### 2.2 Data analysis and study design

CiteSpace, a software tool renowned for visualizing knowledge graphs and analyzing data, provides researchers with insights into network patterns, subject area growth, and citation prominence, thereby elucidating trends in academic research. Meanwhile, VOSviewer, extensively used in library and information sciences, facilitates the visual analysis of literature across various fields. Its subject neutrality and user-friendly interface, combined with a suite of functionalities and advanced visualization features, make it a preferred tool for scholars. VOSviewer (version 1.6.20) enables data manipulation and analysis through label view, density view, cluster density view, and scatter view. In this study, we employed VOSviewer and CiteSpace (version 6.2.R4) to import publications for analysis, examining elements such as titles, keywords, authors, institutions, countries, journals, publication years, citations, average citations, and cited references. The bibliometric data were subsequently exported to Microsoft Excel 2016 to identify publication patterns, document type distribution, and to assess the impact of primary contributors, including authors, institutions, countries, and journals. VOSviewer generated visual representations of connections among these entities, highlighting their scientific influence. Additionally, VOSviewer was used to map co-occurring author keywords, while CiteSpace charted key cited terms, revealing the evolution of knowledge, emerging themes, and potential research frontiers. In the visual map generated by VOSviewer, node colors denote groupings, sizes indicate publication or keyword frequency, links signify collaboration or co-occurrence, and link widths represent strength.

## 3 Results

### 3.1 Trend and annual counts

**Figure 2:**
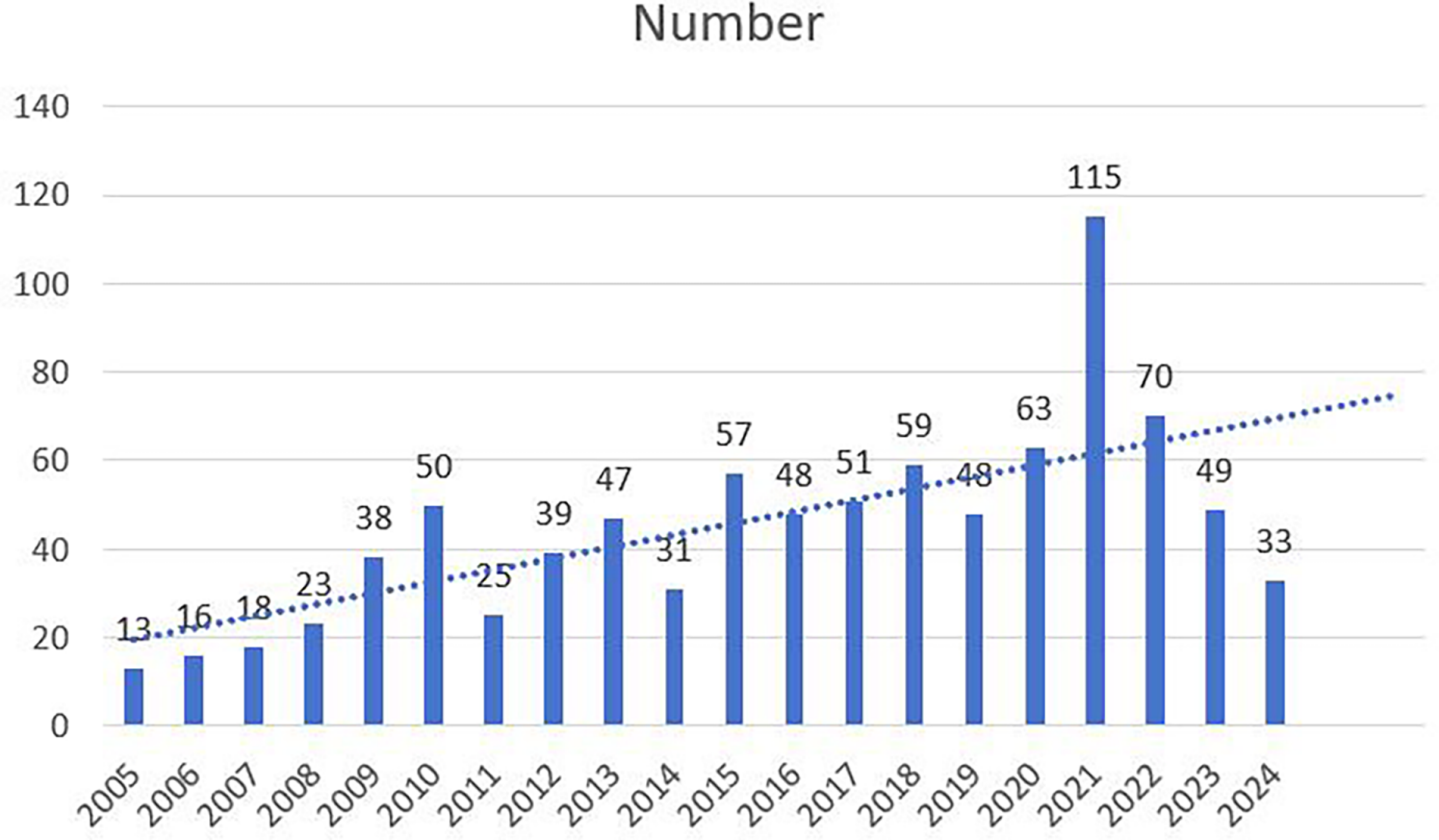
Distribution of publications from 2005 to 2024.

The study examined 893 papers authored by 3,651 individuals affiliated with 1,178 institutions across 68 countries. These papers were published in 67 journals and referenced a total of 15,553 sources from 3,257 distinct journals. The publication period spanned from 2005 to 2024, as illustrated in Figure 2. The dataset comprised 754 articles and 139 reviews, all sourced from public databases and free of any medical ethics concerns.

### 3.2 Analysis of authors

**Figure 3:**
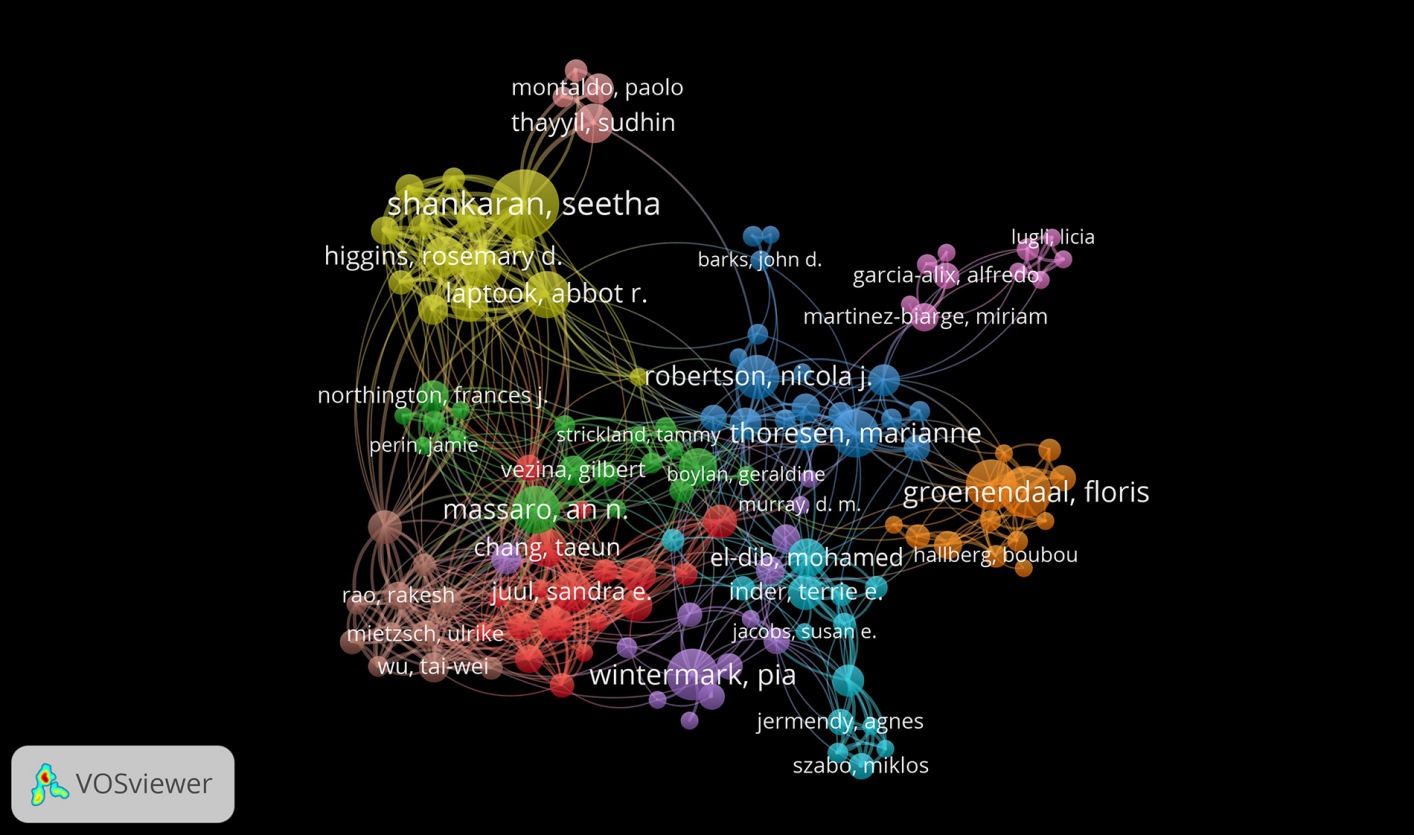
Analysis of authors.

Locat's theorem suggests that around half of the papers in a given field are authored by a highly productive group of individuals (16). This group typically consists of a number of authors equal to approximately the square root of the total number of authors in the field. To determine the minimum number of publications for a core author, one can calculate the square root of the most prolific author's publications and multiply it by 0.749. In this particular field, core authors are expected to have a minimum of 4 publications, with a total of 208 core authors identified. The top five authors, who collectively contributed to 141 papers, represent roughly 15.78% of all publications in the field. Figure 3 illustrates a network visualization depicting collaborative connections among authors. The thickness of the lines signifies the strength of these connections, while the size of the circles denotes the number of articles published by each author. The nodes’ sizes correspond to the number of citations received, and the connecting lines not only link the nodes but also indicate shared reference relationships. The proximity of nodes in the visualization indicates the level of association among authors, often resulting in the formation of distinct clusters. This visualization technique is crucial for aiding researchers in comprehending collaboration and citation relationships, thereby enhancing understanding of academic interactions and partnerships within the scholarly community. Additionally, it offers a unique and user-friendly approach to analyzing intricate relationships within academia. Table 2 highlights the top 5 most influential authors.

**Table 2:** Top 5 most influential authors.

Rank	Author	Documents	Citations	Average Citations/Publication
1	Seetha Shankaran	41	2,008	95
2	Floris Groenendaal	26	974	37
3	Pia Wintermark	26	462	17
4	Linda S de Vries	25	1,561	62
5	Marianne Thoresen	23	1,441	62

Seetha Shankaran has published 41 articles, which have garnered a total of 2,008 citations. Her most cited paper, “Childhood Outcomes After Hypothermia for Neonatal Encephalopathy” (2012), has received 307 citations and examines the effects of therapeutic hypothermia on newborns with hypoxic-ischemic encephalopathy (31). Additionally, her research explores various topics, including brain injury assessment and the long-term outcomes of neonatal encephalopathy (31–33). The majority of her work focuses on the treatment and prognosis of NE, particularly through the application of therapeutic hypothermia (34–36).

Floris Groenendaal is the author of 26 articles, which have collectively received a total of 974 citations. His research emphasizes the importance of imaging assessments in the context of perinatal asphyxia, along with the treatments associated with this condition (37, 38). One notable paper, titled “MR Imaging and Outcome of Term Neonates with Perinatal Asphyxia: Value of Diffusion-Weighted MR Imaging and ^1^H MR Spectroscopy” (2011), has been cited 51 times. This study investigates the relationship between MR imaging and the outcomes of term neonates affected by perinatal asphyxia (39).

Linda S. de Vries has authored 25 articles that have collectively garnered a total of 1,561 citations. Her most cited paper, “Origin and Timing of Brain Lesions in Term Infants with Neonatal Encephalopathy”, has received 144 citations and investigates the causes of neonatal encephalopathy (40). Her research primarily focuses on the assessment of hypothermia in neonatal encephalopathy, the application of imaging techniques in this context, and the perinatal risk factors associated with NE (41–43). Pia Wintermark's research focuses on the incidence and outcomes of neonatal acute kidney injury, the analysis of placental pathology in asphyxiated newborns who are candidates for therapeutic hypothermia, and the utilization of MRI in asphyxiated newborns receiving hypothermia treatment (44–46). One of her prominent works is titled “Placental Pathology in Asphyxiated Newborns Meeting the Criteria for Therapeutic Hypothermia” (2010), which has been cited 42 times (45).

Marianne Thoresen, a distinguished researcher in neonatal medicine, has authored 23 articles that have collectively garnered 1,441 citations. Among her contributions, the study titled “Selective head cooling with mild systemic hypothermia after neonatal encephalopathy: multicentre randomised trial” stands out, with 656 citations (47). This research investigates the efficacy of head cooling in conjunction with mild systemic hypothermia as a treatment for neonatal encephalopathy, illuminating its potential as an intervention for this condition (47). Additionally, Thoresen's work explores the application of moderate hypothermia in managing perinatal asphyxial encephalopathy, providing valuable insights into the therapeutic options available for this disorder (41, 48, 49). Overall, her studies significantly enhance our understanding of the impact of hypothermia on outcomes in cases of NE and perinatal asphyxial encephalopathy, underscoring her expertise in this crucial area of neonatal healthcare (50, 51).

### 3.3 Analysis of country

**Figure 4:**
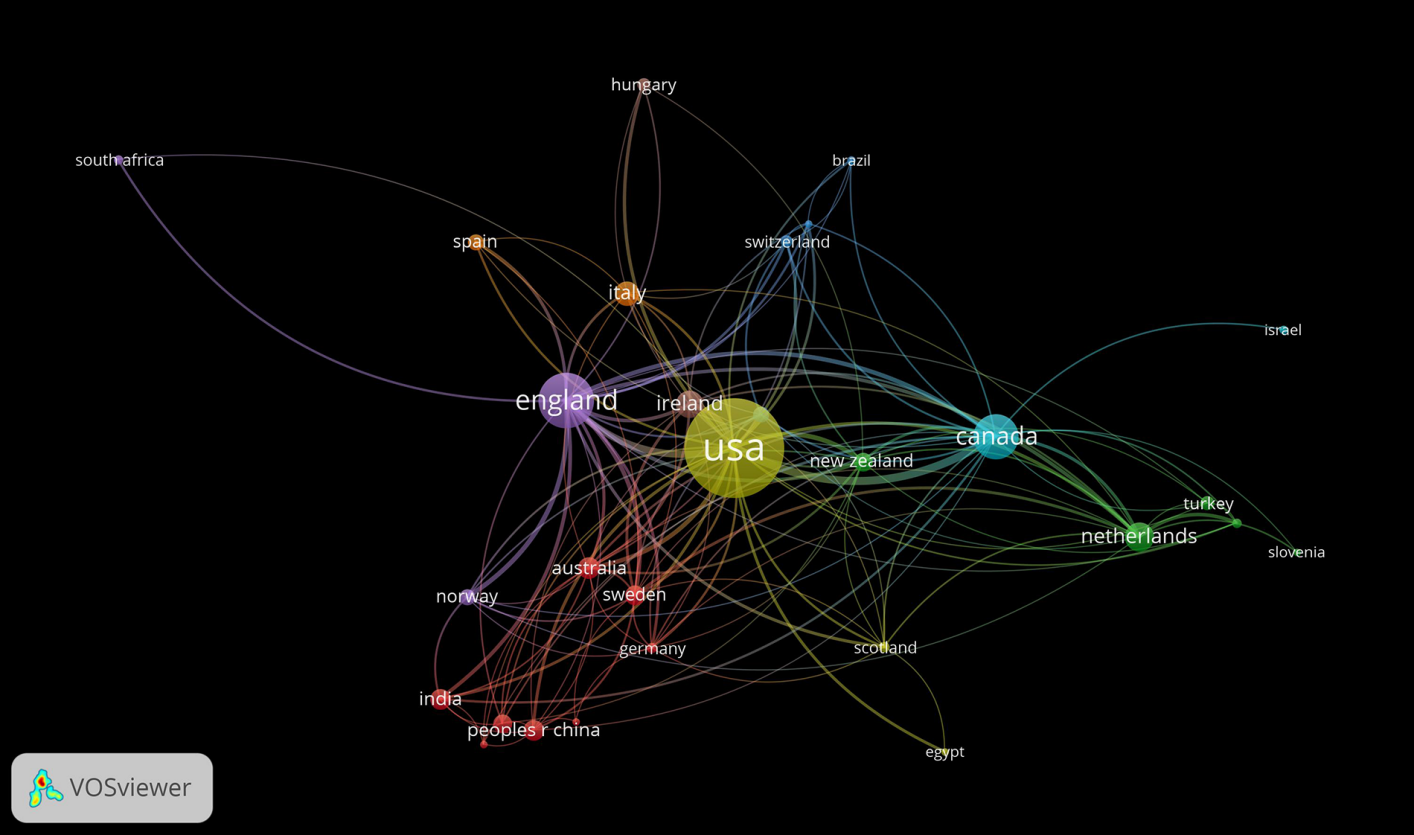
Country collaboration network.

The analysis indicates that research in this field has received contributions from 68 countries, with the United States being the most prolific in terms of publications. The United Kingdom, Canada, the Netherlands, and the Republic of Ireland closely follow in publication numbers. Figure 4 visually represents the collaborative network among these nations, highlighting the top five most influential countries as detailed in Table 3.

**Table 3:** Top 5 most influential countries.

Rank	Country	Documents	Citations	Average Citations/Publication
1	The United States	389	13,888	35
2	The United Kingdom	152	7,306	48
3	Canada	111	4,619	41
4	Netherlands	57	2,968	52
5	Republic of Ireland	51	1,103	21

### 3.4 Analysis of institutions

According to Table 4, the University of Washington has the highest publication count on Neonatal Encephalopathy, with a total of 64 papers. Following closely are Wayne State University with 49 articles and Stanford University with 40 articles. Among the top 10 institutions, University of California, San Francisco leads in citation count, with 3,237 citations, followed by University of Washington, with 3,152 citations, and the University of Bristol with 2,464 citations.

**Table 4:** Top 10 most influential countries.

Rank	Organization	Documents	Citations	Average Citations/Publication
1	University of Washington	64	3,152	49
2	Wayne State University	49	2,253	45
3	Stanford University	40	1,326	33
4	University of California, San Francisco	40	3,237	80
5	McGill University	40	736	18
6	University of Bristol	37	2,464	66
7	University College London	34	1,891	55
8	George Washington University	29	885	30
9	University of Toronto	29	1,330	45
10	Imperial College London	28	862	30

### 3.5 Analysis of keywords

**Figure 5:**
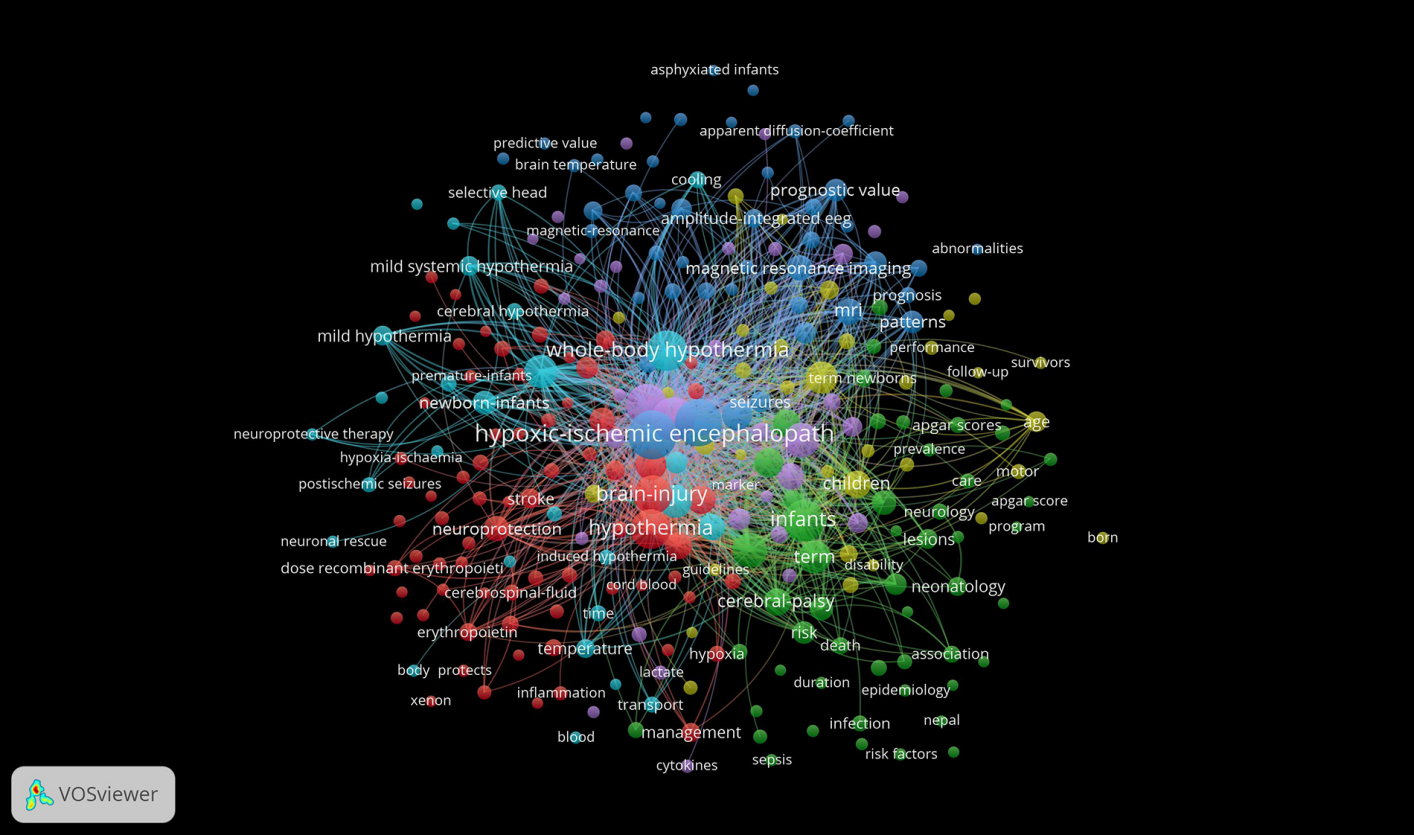
Keywords collaboration network.

**Figure 6:**
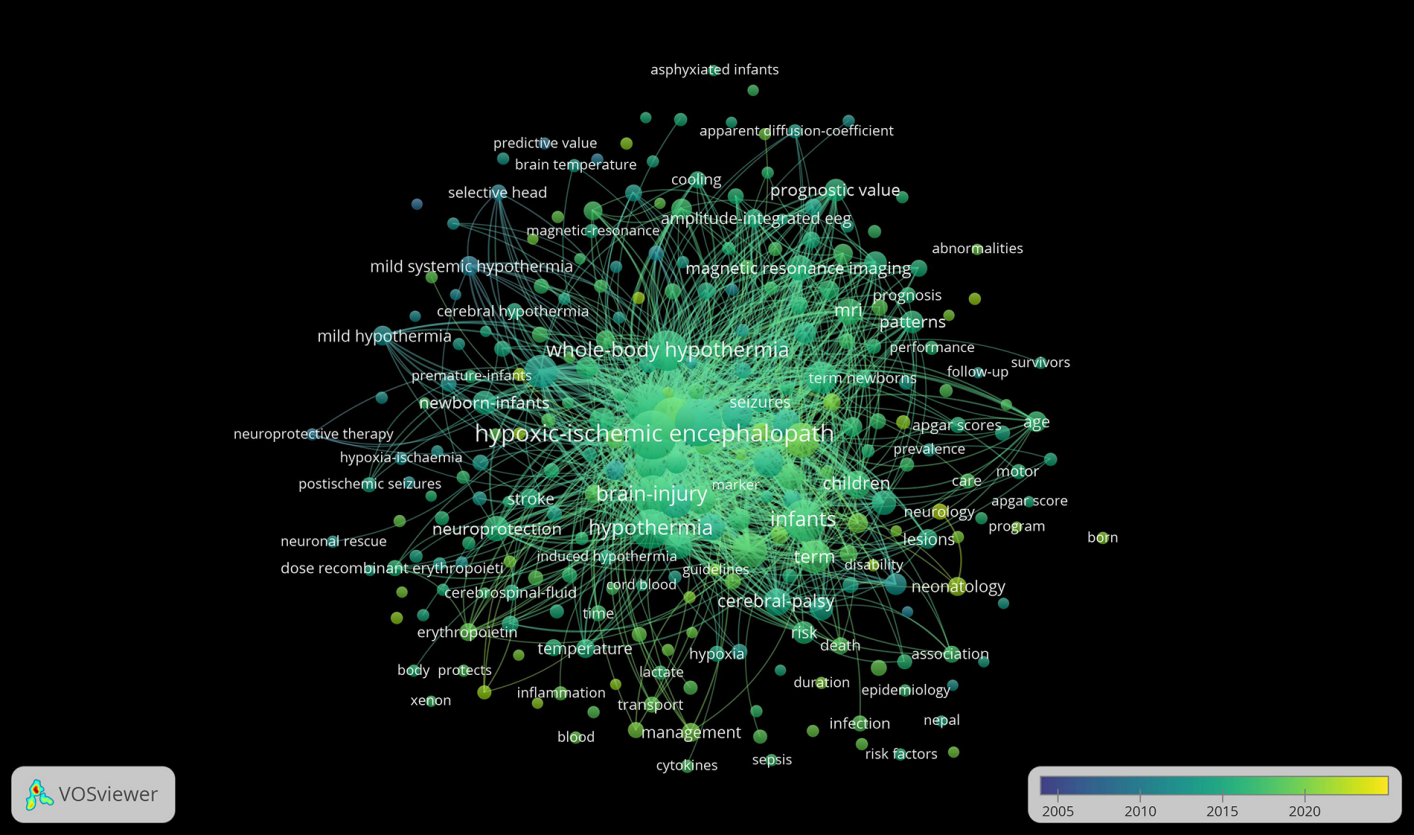
Bibliographic coupling.

**Figure 7:**
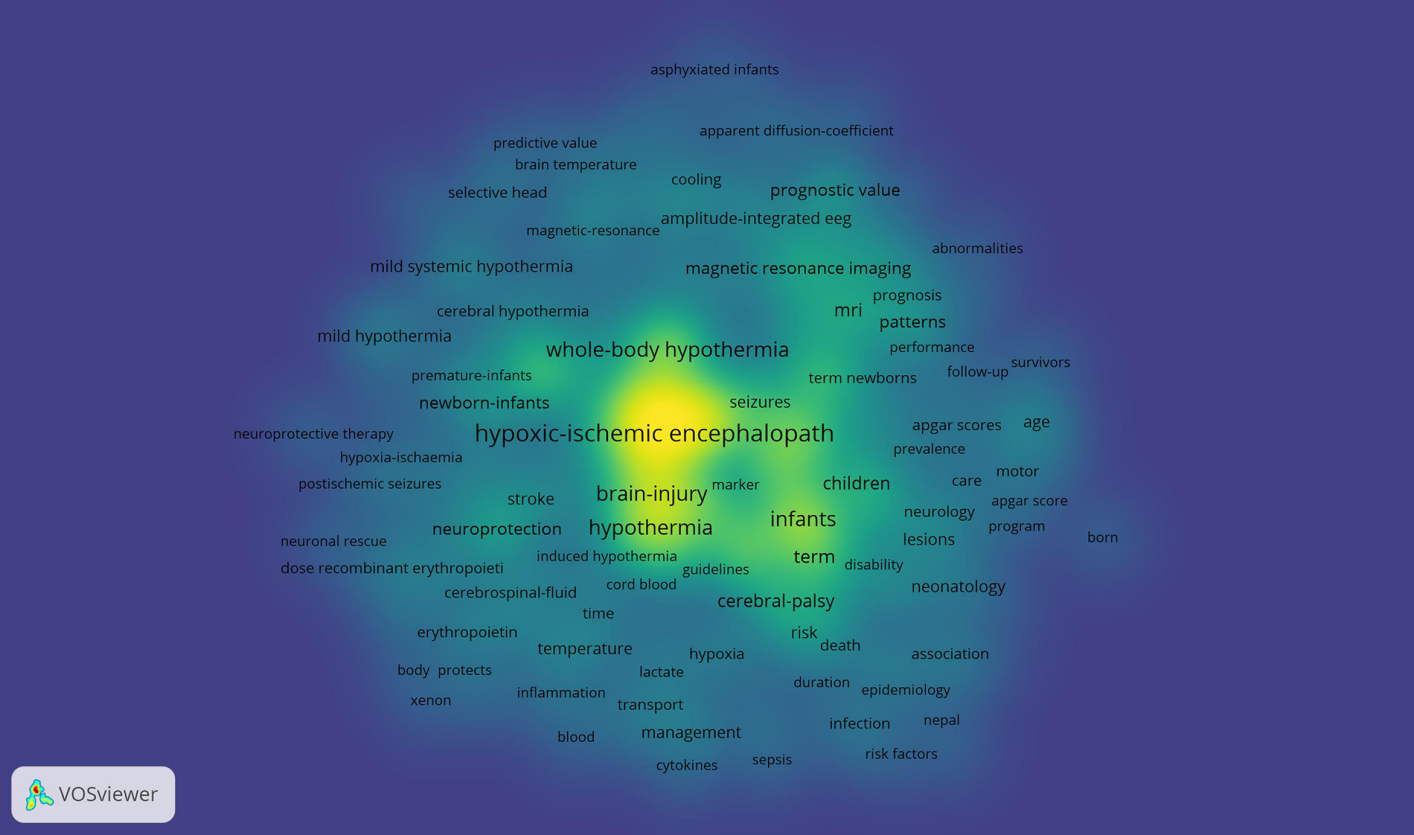
Density map of keywords.

The underlying assumption of co-occurrence analysis is that words that frequently appear together possess thematic connections. VOSviewer visually represents strong relationships between terms by creating distance-based maps, where shorter distances indicate stronger connections. Lines on the map illustrate relationships between items, with more prominent items regarded as more significant within the studied context. Our analysis using VOSviewer concentrated on keywords, as depicted in Figure 5. The clusters, represented in different colors, signify groups of keywords, with node size reflecting their frequency of occurrence. The top ten keywords identified were “Hypoxic-ischemic encephalopathy”, “Neonatal encephalopathy”, “Therapeutic hypothermia”, “Perinatal asphyxia”, “Infants”, “Whole-body hypothermia”, “Hypothermia”, “Brain injury”, “Newborns” and “Outcomes”. Figure 6 presents the bibliographic coupling in an overlay view for articles published from 2005 to 2024, where item size corresponds to the number of citations. Figure 7 illustrates the density map of keywords.

### 3.6 Analysis of journals

**Figure 8:**
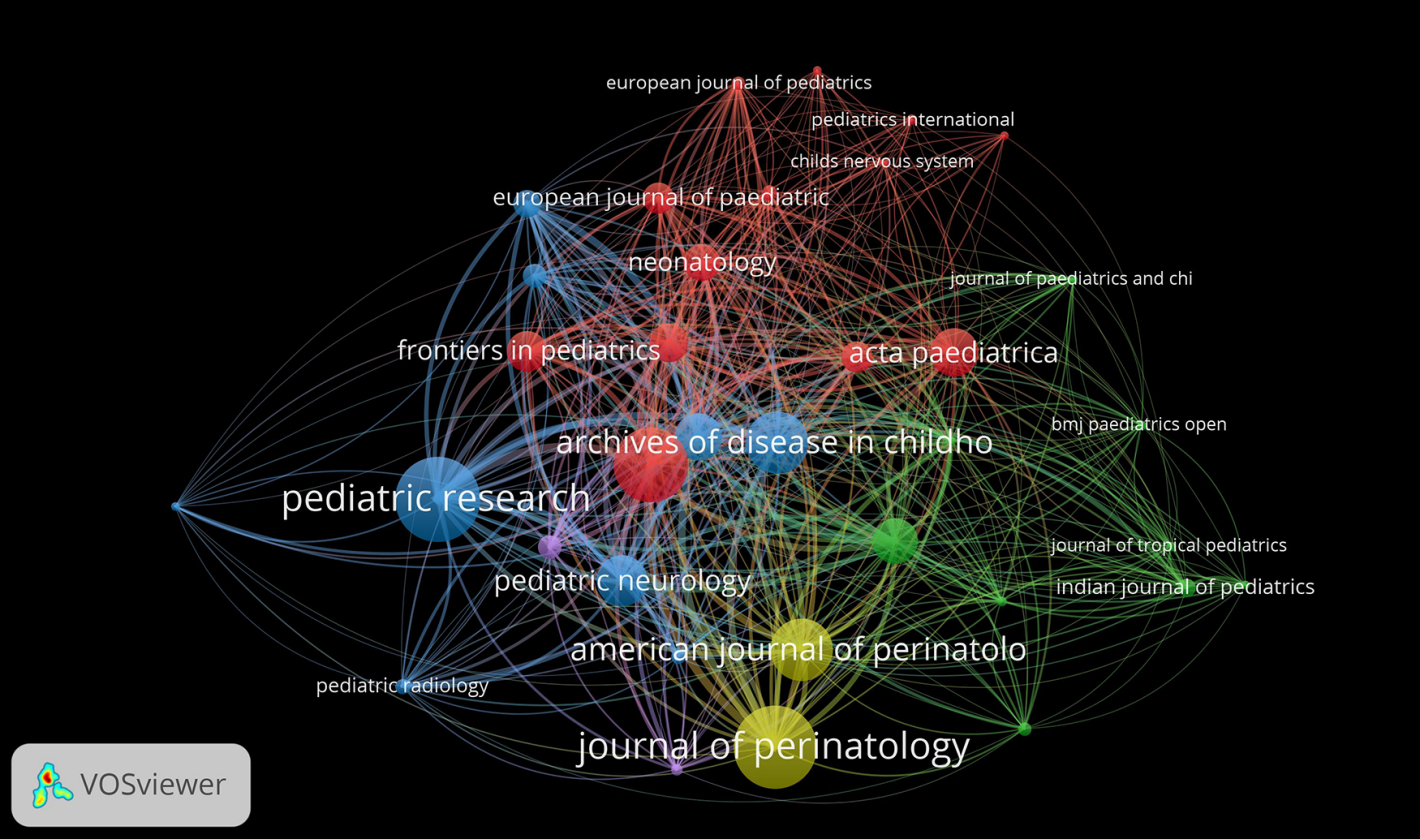
Co-citation analysis network of journals.

Table 5 presents the top ten journals with the highest number of articles on neonatal encephalopathy, with Pediatric Research leading the list by publishing 70 articles. The impact factor serves as a numerical indicator for evaluating a journal's citation rate; notably, Pediatrics boasts the highest impact factor at 6.2, while both the Journal of Pediatrics and the Archives of Disease in Childhood - Fetal and Neonatal Edition each have impact factors of 3.9. By examining co-cited works, researchers can establish a foundational knowledge base for a particular area of study. The forefront of research consists of a selection of referenced publications that contribute to these knowledge foundations. Figure 8 illustrates the connectivity of co-citation among various journals.

**Table 5:** Top 10 most influential journals.

Rank	Journals	IF	Count	Citations
1	Pediatric Research	3.1	70	2,130
2	Journal of Perinatology	2.4	69	1,374
3	Journal of Pediatrics	3.9	62	3,334
4	Archives of Disease in Childhood-Fetal and Neonatal Edition	3.9	52	2,264
5	American Journal of Perinatology	1.5	52	613
6	Pediatric Neurology	3.2	42	1,446
7	Acta Paediatrica	2.4	40	749
8	Pediatrics	6.2	39	4,277
9	Seminars in Fetal and Neonatal Medicine	2.9	38	1,058
10	Frontiers in Pediatrics	2.1	34	457

## 4 Discussion

NE denotes a clinical condition marked by neurological dysfunction, which manifests through a diverse array of symptoms and differing levels of severity. These symptoms may vary from slight irritability and feeding challenges to critical issues such as seizures and coma (52), often emerging in the initial days of life. Additionally, these conditions frequently coincide with breathing difficulties, tone irregularities, and compromised developmental reflexes (53). The study of NE has attracted considerable global attention, underscoring an expanding body of research. An analysis of research papers on neonatal encephalopathy published between 2005 and 2024 indicates a consistent increase in studies over time, reflecting ongoing advancements in this field. Country network analysis revealed that the United States leads in the number of published papers, with all of the top five originating from developed countries. The USA has emerged as a central hub for global collaboration, demonstrating stronger cooperation compared to other nations. Moreover, the USA has outperformed other countries in total publications and citations, suggesting that future research will likely continue to be concentrated in these nations. Co-keyword network analysis identified key research areas within neonatal encephalopathy, emphasizing terms such as “Hypoxic-ischemic encephalopathy”, “Perinatal asphyxia”, and “Therapeutic hypothermia”. These keywords indicate that hypoxic-ischemic encephalopathy, often resulting from perinatal asphyxia, is the most prevalent type of neonatal encephalopathy. Currently, therapeutic hypothermia is considered the most effective treatment for neonatal encephalopathy (54–56).

The University of Washington ranked first in total publications, while Wayne State University, Stanford University led in total citations among research institutions. An analysis of the author collaboration network identified Seetha Shankaran and Floris Groenendaal as the most prolific authors, highlighting their significant influence in the research domain. Pediatrics, which has the highest impact factor, is recognized as a leading journal specializing in neonatal encephalopathy among the top 10 most influential journals.

Extensive randomized clinical studies have consistently illustrated the therapeutic efficacy of hypothermia in neonatal encephalopathy, with methodologies ranging from selective head cooling to the utilization of cooling caps (50, 57–59). The data from clinical trials conducted in wealthier countries indicates that induced hypothermia may have the potential to reduce death or disability in cases of neonatal encephalopathy (11, 60, 61).

The HELIX trial consortium, established in 2010 in partnership with Imperial College London, aimed to conduct comprehensive clinical research on neonatal encephalopathy across South Asia's tertiary neonatal units (62). This trial is distinguished as the largest neonatal cooling study to date, employing a systematic and prospective methodology, utilizing advanced 3 Tesla MRI biomarkers for in-depth analysis, and executed in units with state-of-the-art ventilation technology (63). The trial involved a specialized team of healthcare professionals (63).

The outcomes of the HELIX trial, which focused on hypothermia treatment in low- and middle-income countries, revealed unexpected results. Instead of the expected decrease in death or disability, therapeutic hypothermia was associated with higher mortality rates (63). These findings go against the 2015 guidelines from the International Liaison Committee on Resuscitation, which recommend hypothermia as a standard treatment (64). Systematic reviews have shown mixed results, underscoring the urgent need for alternative treatments that could benefit infants with neonatal encephalopathy in these regions (65, 66). Studies suggest that melatonin may serve as a potential standalone therapy for NE in low- and middle-income countries in the future (67). Additionally, recombinant erythropoietin (rEPO) has been shown to improve both histological and functional outcomes in studies involving neonatal encephalopathy (68). It is likely that future research focusing on melatonin and rEPO for the treatment of neonatal encephalopathy will emerge as a prominent area of study.

NE is primarily observed in sub-Saharan Africa and Southeast Asia (7). It is interesting to note that the top 5 countries leading research on NE are developed nations. This difference could be due to our search strategy being confined to databases like SCI-E, which predominantly feature English publications, potentially introducing bias. Including databases in other languages, such as Chinese or Korean, could yield different results given the large number of papers from East Asia. Bibliometrics, a multidisciplinary field that focuses on the quantitative analysis of scientific literature, can help predict the current status and future trends of NE. However, it may not be able to evaluate the effectiveness of methods using criteria like effect size as seen in meta-analysis. Moreover, bibliometric analysis heavily relies on abstracts, titles, keywords, and references, rather than a thorough examination of the full text, which could potentially impact the final outcomes.

## 5 Conclusion

This research provides a thorough examination and insightful evaluation of literature about neonatal encephalopathy from 2005 to 2024. Using tools such as VOSviewer and CiteSpace, the study explores the current status and changing patterns in this field from multiple perspectives, potentially offering useful perspectives for future research on neonatal encephalopathy.
